# Exploring non-invasive approaches to assist in the detection of clandestine human burials: developing a way forward

**DOI:** 10.1080/20961790.2018.1493809

**Published:** 2019-02-07

**Authors:** Soren Blau, Jon Sterenberg, Patrick Weeden, Fernando Urzedo, Richard Wright, Chris Watson

**Affiliations:** a Victorian Institute of Forensic Medicine, Melbourne, Australia;; b Department of Forensic Medicine, Monash University, Melbourne, Australia;; c Scout Aerial, Brisbane, Queensland, Australia;; d University of Sydney, Sydney, Australia;; e New South Wales (NSW) Office of Environment and Heritage, Sydney, Australia

**Keywords:** Forensic science, forensic anthropology, clandestine graves, mass graves, burial, detection, remote sensing

## Abstract

The analyses of physical evidence recovered from clandestine single and mass graves have been demonstrated to be of significant evidential and/or investigative value for both court purposes and humanitarian investigations. The detection of these types of graves is, therefore, pivotal to forensic investigations. This article reviews different remote and ground-based methods that have been used to attempt to detect deliberately concealed burial sites and summarizes the experimental research that has, to date, been undertaken in order to improve grave detection. The article then presents the preliminary findings of research being undertaken at the Australian Facility for Taphonomic Experimental Research (AFTER). This research, the first of its kind to be undertaken in the southern hemisphere, is based on experimental single and mass graves using human cadavers. The research is centred on current remote sensing methods and techniques combined with the analysis of the effects of below-ground temperature and moisture and ground-based weather data. It is hoped that identifying successful sensors and detectors will be beneficial to national and international agencies that are involved in forensic as well as humanitarian investigations that require the detection of deliberately concealed gravesites.

There is a lack of baseline data about the nature of graves containing buried human remains within different environments to inform methods of detection.— Kathryn J. Powell

## Introduction

Perpetrators of crime involving murder potentially employ a variety of methods, including burial, to dispose of their victims [1]. While haphazard hand dug graves are used to dispose of individual murder victims related to isolated crime [2–6], a variety of grave types have been used by perpetrators of large-scale crimes. Such grave types have been observed in many countries worldwide that have experienced periods of political, ethnic and/or religious violence resulting in the disappearance and deaths of hundreds of thousands of individuals [7–13]. Deceased persons buried in communal graves such as “plague pits”, or graves created following mass disasters [14–16], or used to bury war dead [17] are typically respectfully laid out side by side or head to toe in neat rows. In contrast, multiple or mass graves are often (but not always^1^ [18]) characterized by the random and chaotic placement of multiple bodies. While the exact number of bodies that characterizes a “mass grave” is in dispute [19–21], and there is no definition of the term “mass grave” in international law [22], for the purposes of this research, a mass grave is defined as a “location containing two or more associated bodies” [20].

Haphazard burial of deceased persons may be undertaken due to limited available resources resulting from, for example, ongoing conflict, or as a result of an unfounded fear that mass dead bodies cause major health risks [23]. However, when employed by perpetrators of crime this type of burial highlights a total lack of respect and compassion for the dead by those responsible for the killings [24]. It has also been argued that the magnitude of the crimes represented by a mass grave is much greater than that of a single burial [25,26]. The value of evidentiary and investigative information provided by the scientific investigation of mass graves has been well demonstrated by the results from excavations of mass graves in Latin America in the mid-1980s [27], and subsequently in the mid-1990s in the former Yugoslavia [28,29] and more recently in Spain [30]. The impact of such evidence, whether for court or humanitarian purposes, relies on the success of locating potential sites that may contain graves. However, locating sites may be difficult and complex for a number of reasons: often there is a time lag between the killing event/s and burial and the ability of investigators to gain access to suspect areas. This time lag may be in the magnitude of months or years, with further complications arising from the deliberate concealment of the gravesite location both at the time of grave construction and following burial. Heavily vegetated environments such as forests or jungles may also add to the difficulties associated with locating graves. For this reason “detection of mass graves is at the forefront of international forensics” [31]. Consequently, increasing attention is being given to undertaking research exploring the effectiveness of different detection techniques in a range of environments.

## Methods of detection

Detection of landscape anomalies which may be potential gravesites can be a long, complicated, and often frustrating task. The majority of investigations typically centre on information provided by a witness to the event [32]. If a witness can be located (which is not always possible), his/her statements can lead investigators to a target area [12,33]. However, the process still relies heavily on the witness’s ability to be willing to provide information and for this information to be detailed enough to allow continued investigation. Unfortunately, however, reliance on witness information can be counterproductive in locating the gravesite. Often the time elapsed between the event and investigation can be such that the witness’s memory of events can be vague or even completely inaccurate regarding numbers of individuals and the grave location [34]. Further, the witness may also be one of the perpetrators which adds another dimension to the search for clandestine burials.

Additional but more unusual methods of detection have been employed to target a location. These include dowsing [35,36] and the use of specially trained cadaver dogs [32,37,38]. Based on examples of forensic cases where graves have successfully been located, the most effective detection methods to date involve a variety of land-based geophysical techniques coupled with satellite imagery analysis, landscape survey on foot, and evidence of botanical or geological changes [39–42]. To put all of these into perspective and to explore the potential of the analysis of the landscape, geographical information systems (GIS) have been investigated as a method of targeting potential areas of interest [43,44]. For example, a GIS system was populated and used to narrow potential burial locations within Eastern Bosnia [45]. The results showed that particular information was required prior to any analytical study including variables from previous investigations and excavations [45–47].

While various search techniques have been outlined in detail in numerous text books and journal articles the focus has typically been on the location of single graves [5,48–50]. To date, there has been relatively little published on the use of remote sensing techniques being applied to the location of contemporary multiple or mass gravesites (the exceptions being case studies such as [51–53].

Remote sensing refers to the acquisition of information about an object without physically touching it [55]. Remote sensing technologies have evolved since their initial use in the 1800s from the use of cameras on tethered balloons to photograph the earth’s surface, to the present day use of remote sensors. Imagery and other data for analysis are collected from a range of sensors placed on board aircraft, helicopters, unmanned aerial vehicles (UAVs/drones) and satellites (which provide images used to develop three-dimensional (3D) representations of earth used in geobrowsers such as Google Earth [58]. Remote sensing methods have been employed by the military to map the movement of forces both friendly and aggressive [56], and in areas as diverse as land management, archaeology [57], animal poaching, mining and prospecting. In addition, remote sensing methods have been used to investigate scenes of crime [59], as well as to search for human remains [60,61] and gravesites [45,62,63].

Prior to the 1970s, archaeologists, especially in the UK and Europe, employed the use of aerial imagery to observe crop marks and other subtle variants in images taken of the landscape at height or obliquely to narrow down search areas for potential sites [4,64–66]. The 1980s and 1990s saw an increased employment of remote and newly acquired land-based geophysical techniques and methods [5]. These included the application of ground penetrating radar (GPR) [67] (initially used in the construction industry), and the use of electrical earth resistance [66,68], both which provide additional data on depth and accurate size of target prior to any invasive work being undertaken [4,69,70]. Archaeologists continue to actively apply remote sensing to the search and location of sites of potential interest to span a broad spectrum of time periods from Mayan ruins beneath jungle canopies, Roman cities beneath ploughed fields, and to analyse the battlefields of, for example, the Second World War [71]. These studies have led to a combination of several techniques as the preferred option for many investigations into clandestine burials and mass gravesites.

The potential for remote sensing to be used to identify burial sites was discussed by archaeologists working for the International Criminal Tribunal for the Former Yugoslavia (ICTY) during searches for clandestine graves related to the Bosnian conflicts of the 1990s [72,73]. Many of the larger burial sites had been located using details obtained from ICTY investigations [74] which included statements from witnesses, evidence collected from potential burial locations, and US Government aerial and satellite imagery. Access to this type of imagery allowed forensic teams to identify appropriate personnel and plan for the archaeological and anthropological teams involved in the excavation and recoveries. However, smaller single burial sites could not be located in time for the prosecution to make use of evidence from these related single events [74].

The suggestion of using limited non-invasive geophysics to explore previously investigated or potential sites was realized in 2003 (Sterenberg J., Personal communication, 2017). Combined aerial, satellite and ground-based remote sensing was employed to locate graves from the period that Saddam Hussein was President of Iraq (1979–2003), and provided additional evidence of unlawful killings and mass burials for the Iraqi Special Tribunal (IST) prosecutions [75]. In Iraq, the ability to review images to look for characteristics of a burial prior to dispatching an assessment team to a site enabled military support for operations and security of staff to be fully considered before forensic casework was undertaken. The major disadvantage of these investigations was the heavy reliance on the support and access to technology held by the US Military and its various organizational elements. Despite these restrictions, the initial successes of locating or confirming mass gravesites continued into 2004 with the results indicating that the use of remote spectral analysis in combination with geological assessment in a desert environment could achieve good results for the location of concealed sites [76].

As with all forensic cases, either domestic or international, the advantage of having as much relevant information regarding the possible location and size of the feature (in this case the grave) before excavation commences cannot be underestimated. Even for experienced archaeologists it is often difficult to see the grave-edge, change of context forming the backfill, or possible areas of disturbance. In the context of Iraq, many of the more politically sensitive sites were alleged to be located in the south-west of the country in a desert environment making assessment difficult [77]. Weather conditions and landscape changes also caused several reported sites to become virtually invisible over time including many of the sites dating to the early to late 1980s. Due to the overwhelming number of reported sites (which at one stage numbered over 270), the Coalition Provisional Authority's (CPA) (US Government) forensic archaeological team employed a searchable database of basic information on location, size and possible victims. Constructed from several sources including Criminal Investigation Division (CID) (US Army), Human Rights Watch groups and witness statements, this database was further supplemented by the US Army who provided unclassified images of the locations in question. The use of all forms of available information allowed spectral analysis to be undertaken on those sites that were of particular interest to the ongoing establishment of the Regime Crimes Liaison Office and the IST [76,78].

A similar combined approach was undertaken in 2005 to identify potential unexcavated mass gravesites in eastern Bosnia [40,73]. A team of scientists combined commercial satellite imagery, limited spectral analysis, a purpose-built GIS system looking at specific variables [79], electrical resistivity, entomology, soil analysis, and the specifics of imported or foreign flora and fauna to produce accurate 3D models of potential mass graves and their contents. A grave was located and excavated to compare results of the resistivity images of possible body mass and the grave dimensions, which were found to be exact matches [80]. The results appeared to once again indicate that a variety of methods and techniques could be integrated to improve locational work. However, lack of additional funding meant that it was not possible to continue to the next step; the use of drone-mounted survey techniques.

Anecdotal evidence from forensic archaeologists and anthropologists working on locating clandestine burials sites in diverse environments including the European landscape of the Caucasus, the deserts of Iraq and Kuwait, and heavily forested sections of Mexico, stresses that difficulties in locating gravesites continue to bring frustration to all those involved in excavation, recovery and repatriation work. Complex topography and dense vegetation cover can mean that access to burial sites and/or mass gravesites can be challenging [81,82]. The increasing need to consider alternative strategies that include remotely piloted aircraft systems to gather data when access is limited, and broader areas need to be investigated are highlighted by the potential dangers involved with investigating graves “on the ground” as demonstrated by the beheading of UN investigators in 2017 in the Democratic Republic of Congo when they travelled to Bukonde to search for mass graves [83].

## Research to improve practise

Research undertaken to improve the detection of clandestine graves has, to date, predominantly focused on single burials and has typically used non-human (animal) carcasses as human analogues with particular focus on one technique or method (Table 1). Similar to research carried out to improve the ability to locate single graves, research undertaken on mass graves has predominantly involved animal models (Table 2). The effects of decomposition when there is more than one individual in a grave have been investigated using mice [84] and rabbits [85]. Animal models have also been used to investigate different search techniques.

**Table 1.** Summary of research using experimental single graves to test various detection techniques.

Construction method	Contents of individual graves	Other items	Depth	Environment/location	Time between burial and search	Search method	References
Hand (no detail provided)	6 pigs	No	50.8–78.7 cm	Bush covered/Colorado, USA	1 month–7 years	Magnetics, electromagnetics, GPR, cadaver dogs, thermal imaging, aerial photography	[49,86,87]
Hand (shovels and picks)	3 kangaroos	No	1 m	Arid/semi-arid/Adelaide, South Australia	4 years	GPR	[88]
Hand (shovels and picks)	3 pigs	No	0.5 m	Arid/semi-arid/Adelaide, South Australia	8 months	GPR	[88]
Not described	12 pigs	No	6 buried shallow (0.5–0.6 m) 6 buried deep (1.00–1.10 m)	Open field away from tress and fences: Ultisol soil type consisting of sand and clay horizons	13 months, 21 months, 21.5 months	GPR	[89]
Not described	12 pigs	No	6 buried shallow (0.5–0.6 m) 6 buried deep (1.00–1.10 m)	Open field away from tress and fences: Ultisol soil type consisting of sand and clay horizons	13 months, 13.25 months, 21 months	GPR	[90]
Hand		3 pigs 1 naked; 1 wrapped in tarpaulin	0.5 m	Grassed area/Staffordshire, UK	0–3 years; 4–6 years	Electrical resistivity and GPR	[64,91]
Hand	1 plastic resin skeleton with animal soft tissue and salt solution	Clothed	0.6 m	“Made-ground” clay-rich soil type/Staffordshire, UK	1 month	Magnetic susceptibility	[92]
Hand	1 metal-jointed fibreglass mannequin	Clothed	0.5 m	Sand dunes/north-west England, UK	Not provided	Magnetic susceptibility	[92]
Hand	1 pig, 1 control	No detail	No detail	Mixed-wood forest and clearings/semi-urban outskirts of Ottawa, Canada	Not provided	Ground-based and aerial hyperspectral	[93]

GPR: ground penetrating radar

**Table 2.** Summary of research using experimental mass graves to test various detection techniques.

Construction method	Contents of mass graves	Other items	Dimensions	Environment/ location	Time between burial and search/recovery	Search method	References
Motorized backhoe attached to a caterpillar tractor	8 juvenile cattles laid out head to head	Blunt force trauma to the head from a mallet and a bullet to the back of the head; unclothed	5 m × 5 m × 1.5 m Control	Transitional tropical moist forest/Costa Rica	5 months	Spectral measurements using both *in situ* reflectance measurements and hyperspectral analysis (airborne imagery)	[94]
Hand	2 mice (placed horizontally, side by side), 5 mice (stacked in a pyramid), 10 mice (placed haphazardly in a pile)	Mice placed within a wooden bookcase; each mouse wrapped in cotton to simulate clothing	127 cm × 123 cm × 30.5 cm	Yellow brown, sandy clay/Backyard, Oyster Bay, New York	1 week, 2 weeks, 1 month, 2 months	Not provided	[84]
Hand	21 rabbits in 10 graves (5 columns and 2 rows)	Gunshot wounds	60 cm × 60 cm × 60 cm	Rough pastureland surrounded by a thin mixed native tree line/northwest England, Central Lancashire’s Taphonomic Research in Anthropology, UK	Every 10 days, up to 60 days	Not provided	[85]
Machine (no details)	Cows	No detail	No details	Temperate environment/UK	10 years; 18 days repeat period or multiple thereof	Multi-spectral, fine spatial-resolution satellite imagery	[31]

Some burials have been deliberately altered in order to assess the effects on spectral imaging including the covering of the remains with fertilizer treatments such as manure, blood and bone meal [95]. A range of different geophysical techniques has been tested with varying results in different environments. These techniques include GPR with different frequencies of antennae [49,96], electrical resistivity including longline surveys [64,91] and magnetic susceptibility [92]. More recent research using single burials has investigated the use of remote hyperspectral imaging as a potential search tool [93] (Tables 1 and 2).

Currently, there is no technique that will identify the presence of a body (single or multiple) within a grave. Anomalies, however, can be detected all of which require excavation to confirm or eliminate them from further enquiry [97]. In certain geological environments, earth resistivity can be rapidly undertaken and produce good results for single burials associated with coffins (e.g. historic cemeteries with unmarked graves) [98–100] and larger more complex features such as mass graves (see above). GPR [101] and magnetic susceptibility [92] can also provide indicators of single burials, however, the survey results need to be carefully analysed by an experienced operator.

In 2003, Jessee [25] highlighted the need for research to attempt to improve the detection of graves, in particular through the creation of a series of experimental mass graves and mass grave-related test sites. Such research requires multiple human cadavers (and therefore appropriate ethics approval), as well as access to secure research facilities big enough to dig large holes. While research involving graves may be undertaken in university facilities it was predicted that funding pressures associated with timely presentation of results may hinder the ability to leave remains in the ground for significant amounts of time. Consequently, there has been the development of taphonomic research facilities that use donated human bodies: seven in the United States, one being developed in the Netherlands and one in Australia [54].

Much of the research on the taphonomic changes and effects of decomposition within large multiple and mass graves has been based on the work of forensic pathologist Dr. Arthur Mant. At the end of the Second World War in Europe Mant was tasked to record several mass graves as a means of retrieving evidence of war crimes and crimes against humanity. His work scientifically proved that a mass grave constitutes its own distinct microenvironment [102,103]. These early observations were affirmed during the recoveries from large-scale forensic investigations undertaken in the 1990s and into the 2000s in the former Yugoslavia [104]. The vast number of differentially preserved remains being recovered allowed scientists to ascertain that there were differential decay rates in single, multiple and mass graves [104], and that subtle changes in decomposition could ultimately be affected by burial environment (e.g. depth, number of remains, compaction) and soil type (sand, clay, loam) and condition (waterlogged, etc.) [105,106].

Such observations indicated that if these variables could be scientifically monitored it may be possible to employ modern technological advances to undertake the following:

- Detect indicators of clandestine burial or grave disturbance;
- Establish a time between the excavation and backfilling of a burial or grave and establish a time frame where it is still detectable;
- Establish possible further identifiable effects of the multitude of intrinsic and extrinsic variables of decomposition on detection; and/or
- Determine whether the decomposition of more than one individual can have a visible and discernable effect on detection techniques.

In 1981, research involving human cadavers began at the Anthropology Research Facility at the University of Tennessee [54]. Initial research projects involving human remains focused on the decomposition of single human cadavers buried and placed on the surface. Subsequent projects involved testing different search/detection techniques for buried remains (e.g. in Australia [88] and Colombia [107,108]. The limitations of this initial work included certain variables that did not replicate a real-world situation, for example, the inclusion of a wire cage over the gravesites to limit scavenging [88], and the burial of already skeletonized and burnt remains [107,108].

In February 2013, the first use of human cadavers specifically being used in mass grave research commenced at the Anthropology Research Facility at the University of Tennessee [109]. Four hand-excavated graves were created: one grave containing six individuals, one with three individuals, and another with a single individual. An additional grave was dug out and refilled but was left empty in order to act as a control [110]. Since these initial burials, an additional grave containing 24 individuals has also been created (Mundorff AZ. Personal communication, 2017).

While there are several taphonomic research facilities that use donated human bodies [54], all of these facilities are in the northern hemisphere. In January 2016, the Australian Facility for Taphonomic Experimental Research (AFTER) was established by the University of Technology, Sydney (UTS). This facility is the first of its kind in the southern hemisphere and provides the opportunity to develop research and collaboration between institutions undertaking research on the detection of single and mass human burials in different environments.

In July 2016, ethics approval was provided by the UTS to undertake a project which aims to document in detail single and mass graves in a specific southern hemisphere context. The research is being undertaken over a period of at least 3 years providing a more in-depth understanding of decomposition processes and the impacts on mass grave detection methods. The premise of the research design is based on scenarios observed around the globe where victims of ongoing conflicts are buried within small or large mass graves. In addition, a separate individual burial was created as a possible scenario to improve current police investigations.

### Methodology

**Figure 1.**
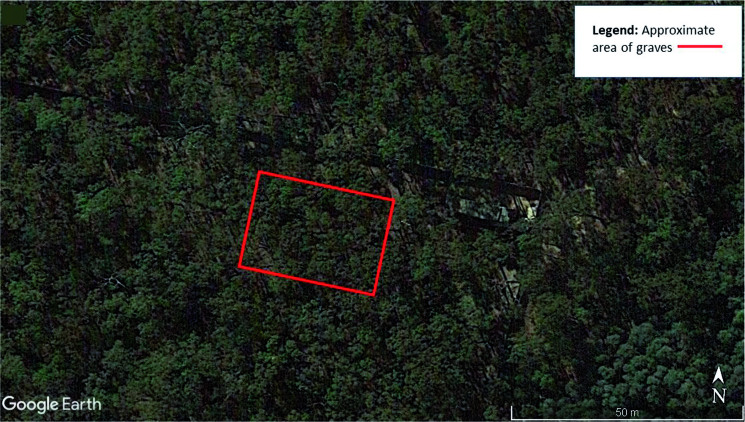
Aerial view of the Australian Facility for Taphonomic Experimental Research (AFTER) facility indicating area of research. Google earth pro version 7.3. (May 5, 2016). Yarramundi, New South Wales, Australia 33°38'51.38''S and 150°39'46.84''E, Eye alt 500 m. Map data: Google, Landsat/Copernicus 2018. http://www.earth.google.com (May 10, 2018).

An area of land measuring 30 m × 30 m at the AFTER facility (a site which encompasses approximately 0.0486  km^2^ of land) was chosen for the research to be undertaken. Located close to a dirt track, the area allowed machine access to excavate and backfill graves whilst still maintaining constant 24 h monitoring of any relevant surface change in the area to be monitored over a 3-year period. The site is located within a Cumberland dry sclerophyll forest (eucalypt woodland). The natural overhead tree canopy creates a relatively dense but even coverage (Figure 1).

**Figure 2.**
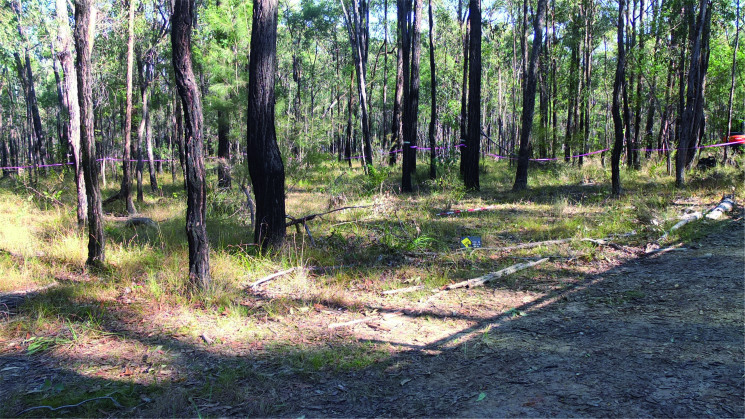
View (looking south–west) of the area designated for the experimental graves. NB: the cleared area to the right of the image is the dirt track.

The area was chosen for the construction of the graves slopes gently from north to south with partial cover provided by vegetation of different strata (Figure 2). The underlying geology was identified as sandy silts with variable quantities of clay (A and B soil horizons), forming above a highly weathered bedrock (saprolite) layer overlying shale/siltstone/sandstone bedrock with a pH range from 5.5 to 6.5 [111]. Prior to any invasive excavation to create the graves a GPR survey was undertaken by staff from the Australian Federal Police (AFP) which acted as a control for subsequent GPR surveys.

**Figure 3.**
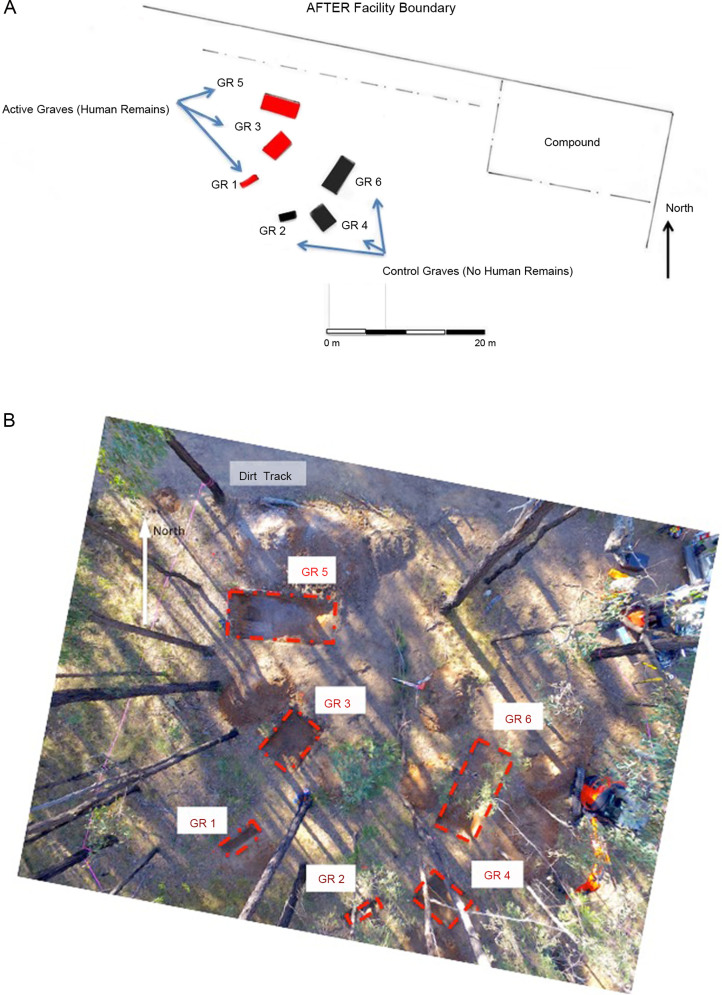
Plan (A) and aerial photograph (B) of the six experimental graves (GR1–GR6) at Australian Facility for Taphonomic Experimental Research (AFTER).

**Figure 4.**
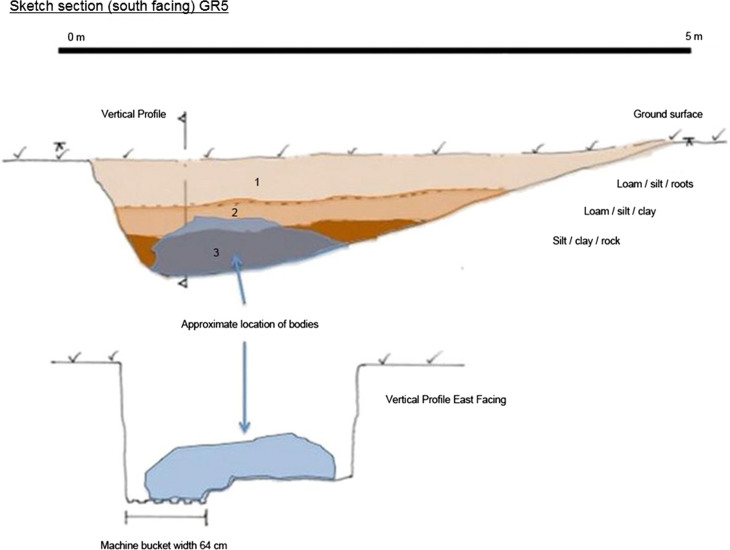
GR5 section and profile.

**Figure 5.**
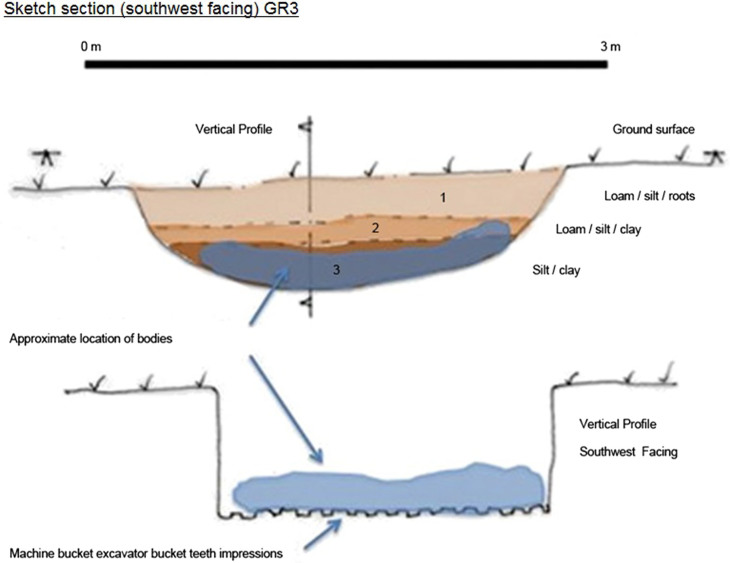
GR3 section and profile.

**Figure 6.**
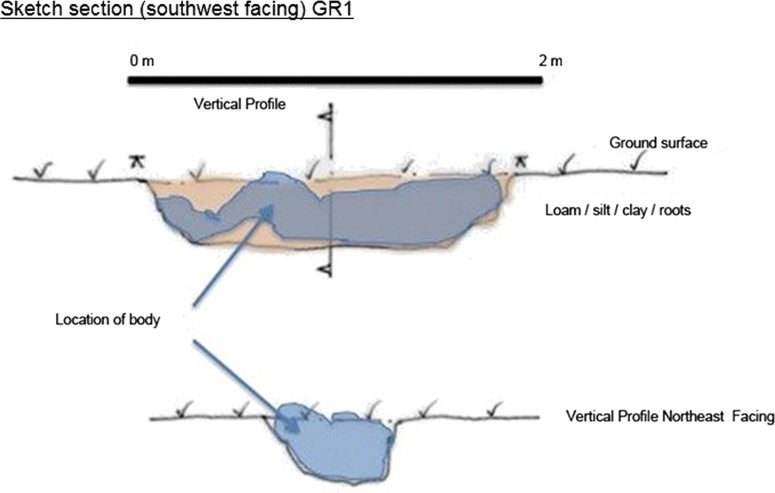
GR1 section and profile.

A total of six experimental graves were prepared (Table 3, Figure 3). A small 5-tonne rubber tracked excavator with a toothed “general excavation” bucket (0.64 m width) was used to construct the large mass grave, Grave 5 (GR5) (Figure 4), and the smaller multiple Grave 3 (GR3) (Figure 5). The single burial, Grave 1 (GR1) (Figure 6) was excavated and backfilled using hand tools. Each grave was excavated to various depths in order to see if depth as a variable potentially influences detection when there are no bodies (control graves) compared depth those graves with bodies. To eliminate any potential leeching of decomposition fluids between graves a distance of approximately 4 m was left between the graves containing human remains (active) with a distance of approximately 10 m between active and the duplicate empty control graves (GR2, GR4 and GR6). Due to the stability of the underlying geology, there were no perceived problems with drainage issues.

**Table 3.** Summary of the experimental graves created at Australian Facility for Taphonomic Experimental Research (AFTER).

Grave	Number of individuals	Method of excavation	Dimensions
GR1	1	Hand	2 m × 0.3 m × 0.3 m; slight slope to each end and slight slope along east edge, flat base
GR2	0 (control)	Hand	2 m ×0.3 m ×0.3 m
GR3	3	Machine	3 m × 2 m × 1 m; consistent level base from side to side, vertical edges, sloped from centre to upper edge
GR4	0 (control)	Machine	3 m × 2 m × 1 m
GR5	6	Machine	5 m × 2 m × 1.4 m (at the deepest end to the west); consistent level across base, vertical sides, slight slope west end of grave from base to ground surface, long gentle slope “ramp surface” from deepest point to ground surface east end of grave
GR6	0 (control)	Machine	5 m × 2 m × 1.4 m (at the deepest end)

To populate graves GR1, GR3 and GR5, 10 donated human cadavers were made available for the research by the UTS. Prior to interment, each donor was scanned using computed tomography (CT) in order to have a permanent record of soft and hard tissue prior to burial. In addition, a range of data was collected for each individual including height, weight, date of death, cause of death, period in storage, body storage temperature, time between storage and burial (thaw), place of birth and place of residence in the last 10 years (Table 4). These data will be reviewed following the exhumations in order to interpret decomposition and the possible effects this may have on the detection of the graves.

**Table 4.** Summary of information about the human cadavers used in the experimental research.

Body #	Grave	Sex	Age (year)	Height (cm)	Clothed	Footwear
B01	GR1	M	77	172	Yes	Socks and shoes
B02	GR3	M	85	165	No	No
B03	GR3	F	82	154	Yes	Sandals
B04	GR3	F	75	172	Yes	Sandals
B05	GR5	F	67	152	No	No
B06	GR5	M	62	164	Yes	Sandals
B07	GR5	F	74	147	Yes	Sandals
B08	GR5	F	58	165	Yes	Crocs™
B09	GR5	M	55	182	Yes	Thongs
B10	GR5	M	69	171	Yes	No

Each individual was photographed and two DNA samples (buccal swab and toenail) were collected. Samples of hair from each individual were also collected. The majority of individuals were dressed in a combination of natural and artificial fabrics and were buried with a range of artefacts including a mobile phone, projectiles and shell cases. While it was not possible to traumatize the individuals, a number of the shirts were shot using a 9 mm Glock 22 pistol in order to create gunshot residue (GSR). Two individuals (one in GR3 and another in GR5) were buried without clothing. These data will form the basis of additional projects examining the preservation of different types of evidence in single and mass graves.

**Figure 7.**
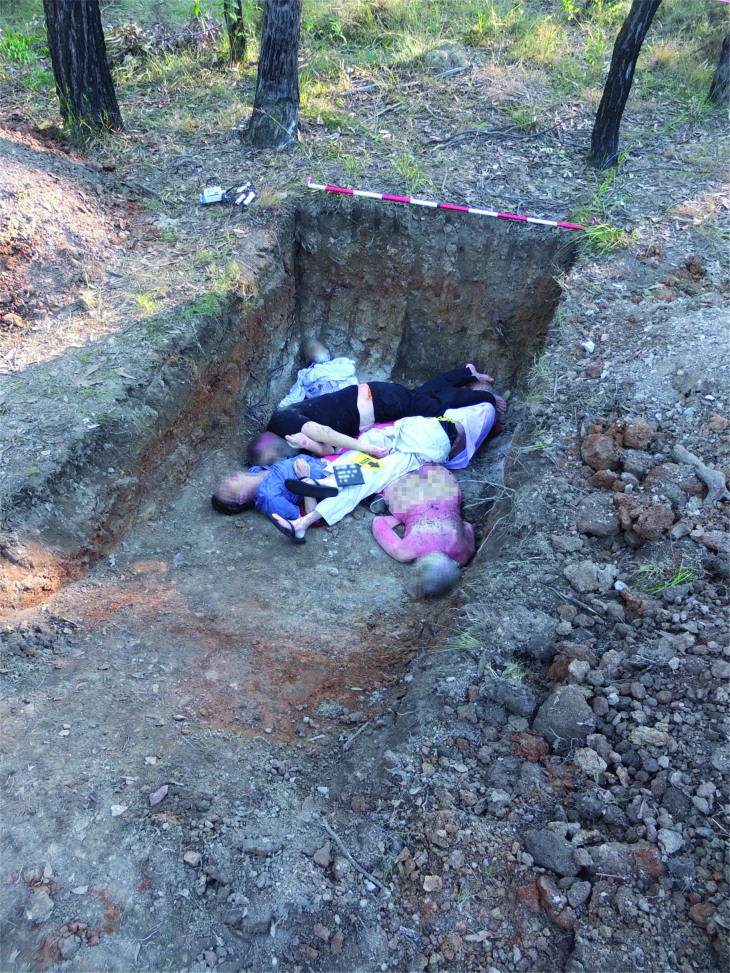
Human cadavers in GR5 prior to placement of temperature and moisture data loggers and backfilling.

GR5 contained six individuals who were placed in a mounded haphazard manner and grouped at the deepest end of the grave. It should be noted that unlike previous mass grave studies which have been undertaken in Columbia, the UK and the US where remains have been laid out in rows (often head to toe), the individuals within the graves at AFTER were specifically placed in positions to represent an alternative layout as observed in many clandestine mass graves (Figure 7).

A digital image record of all of the individuals was completed prior to burial together with a 3D image scan undertaken by the AFP once placed in their final location. The AFP also undertook vertical drone imaging from a variety of heights of each gravesite during the construction and backfilling process.

To record temperature and moisture changes electronic data loggers were placed within the graves prior to backfilling. Seven temperature and four moisture loggers were placed under or in close proximity to individual cadavers. All of the graves containing bodies had temperature and moisture loggers placed in the graves with the larger of the graves (GR5) containing two temperature loggers (RX3003 3G remote monitoring station with RX3000 Hobo Temperature Probe, MicroDAQ.COM.LTD, Contoocook, NH, USA), one within the body mass and the other towards the eastern aspect of the grave towards the ramp. The moisture logger (S-SMA-005 Smart Soil Moisture Sensor, MicroDAQ.COM.LTD) was placed within the body mass. The data cables were placed on the edges of each grave and ran to monitoring stations above ground allowing periodic maintenance to be undertaken when necessary. These loggers allowed the constant recording of moisture and temperature data over time.

Monitoring of entomological information began as soon as the first individual was placed within a grave with the various forms of insects (including different species of flies and ants) noted as and when they began to locate the cadavers. Placement of all individual cadavers was undertaken over a period of approximately 8 h (which included the time of transport of the bodies to the site, collection of DNA samples, dressing the individuals and finally placing them in the graves). The six graves were then backfilled: GR1 and GR2 by hand and the remainder by machine. The backfill was compacted to a stable level by the machine excavator and left as an indicator of potential concealment. A second GPR survey was then undertaken by AFP using a series of control points for scans across the graves. These control points and initial scans will be used for future reference. A total of four GPR surveys have been undertaken to date. The results of these surveys will be presented separately.

## Data collection

### Data loggers

The temperature and moisture loggers were activated at 1:00 p.m. on Thursday, June 17 2016. Data from the loggers were recorded every hour and collected by a member of UTS staff on a fortnightly basis. Additional above ground data from a permanent weather station within the AFTER facility were also collected, both sets of data being used for analysis.

### Time-lapse cameras

A dedicated time-lapse camera was mounted at the site. This camera specifically focused on the larger mass grave (GR5). The unit was capable of recording image files as a single time-lapse video per day with one image taken every 10 min. This rate was higher during the first week in order to take in the initial creation of the graves and any significant backfilling episodes. The camera footage was used to record changes within the backfill of the grave.

### Aerial survey

An initial aerial survey of approximately 0.02 km^2^ (incorporating the grave site) was undertaken in June 2016. Lightweight drones equipped with multispectral sensors flew at a height of approximately 40 m above ground level capturing reflected light at visible (RGB), Green (550 nm), Red (660 nm), Red Edge (735 nm) and near infra-red (790 nm) spectra. The data gathered from this survey were processed by specialized photogrammetric software to generate reflectance maps and a digital elevation model (DEM), which formed the base for future airborne light detection and ranging (LiDAR) or 3D laser scanning surveys [112].

LiDAR involves the use of light sensors to measure the distance between the sensors and the target object (in this case, the ground) and calculating distance by measuring the time it takes for a reflected signal to return [113]. Drone flights were executed using multirotor and fixed-wing aircrafts over the area of interest. Flight approval was provided by the nearby Royal Australian Air Force (RAAF) air base at Richmond. Additional airborne LiDAR surveys were subsequently conducted at AFTER on May 18 and October 24, 2017 to record any significant changes in the area of the graves. In order to ensure an unbiased approach to the analyses, the data obtained from the LiDAR survey were analysed by a specialist who had not visited the location (i.e. was unfamiliar with the landscape) and was not provided with the details of the burials (i.e. the number of bodies in each grave).

### Botanical survey

A total of five botanical surveys were completed. The species present on both the grave and the spoil for “grave” and “control” at each location were recorded including an estimation of vegetation cover for different species. General observation of the gravesites and the surrounding vegetation (e.g. vegetation cover of the surrounds and species phenophase) was also recorded to enable subsequent analysis of vegetation succession on the disturbed areas and identification of any foreign or indicator flora species.

### Soil analysis

Initial soil descriptions were completed prior to intrusive work from the open graves sections. These included initial collection of soil samples to monitor possible detectable chemical changes within the backfill of the graves. While additional soil sampling was planned, due to the compact nature of the soil within the backfill (dry and/or too coarse for the biopsy needle), the soil was only sampled to a depth of 5 cm with a 15 mL vial. It is unknown at this stage of the research whether longer and stronger probes can overcome this problem. Each probe hole was plugged and marked (using a green plastic peg) following sampling.

## Preliminary results

The following information is a summary of the preliminary results gathered between June 2016 and October 2017 with the main focus of this article being analysis of data from GR5, the mass grave containing six individuals. It should be noted that this research is ongoing and as such variants within the data may occur. The results are described before and after placement of human remains in the graves and the creation of the controls (June 2016), and the first (May 2017) and second (October 2017) airborne LiDAR surveys.

### Temperature and moisture

**Figure 8.**
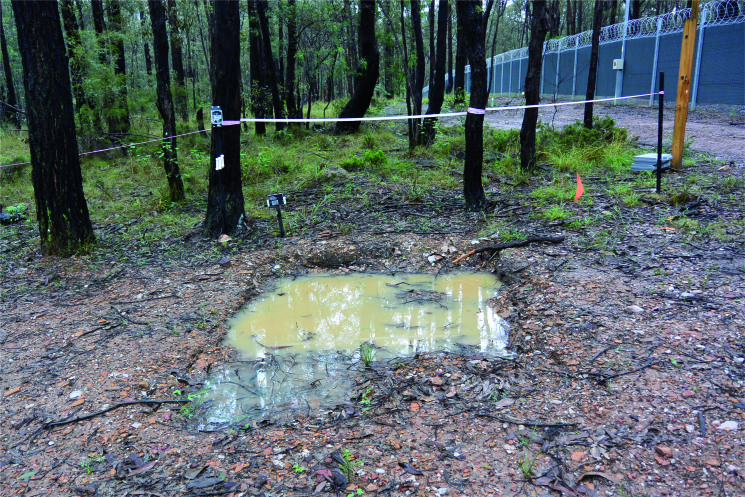
GR5 looking west showing obvious pooling of water, March 2017.

The experimental graves were created in winter (June 2016) where the temperatures were typical of an eastern Australian (Sydney) winter. Average winter temperatures range from 8 °C (46 °F) to 16 °C (61 °F) (in July), as compared to the summer temperatures in the Sydney region which range from 19 °C (66 °F) to 26 °C (79 °F) (in January). The average rainfall for the month of February 2017 was recorded by the Australian Bureau of Meteorology at Richmond RAAF airbase as 32.6 mm. However, March 2017 experienced significant rainfall across the region with one of the wettest months of March on record [114]. Pooling of water was visible in GR5 during March 2017 (Figure 8).

**Figure 9.**
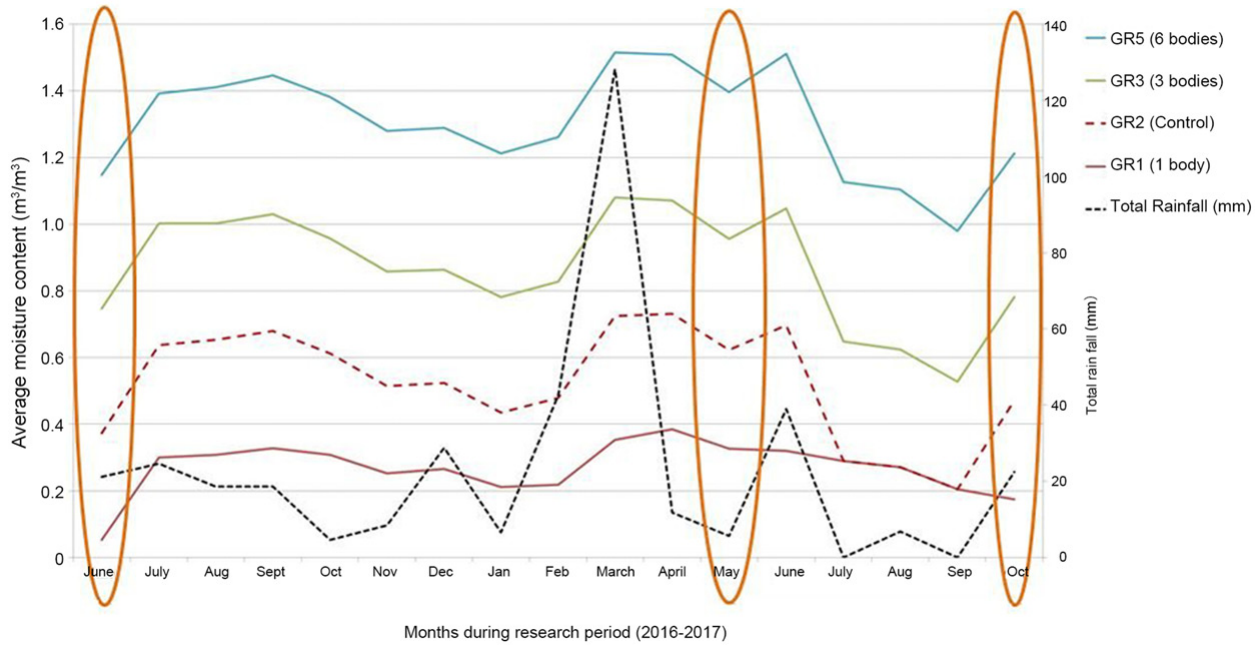
Total monthly rainfall (June 2016–October 2017) compared to the average monthly moisture content for GR1, GR2, GR3 and GR5. NB: Orange ovals indicate the months when the LiDAR surveys were completed.

Despite this increased precipitation in March 2017, the moisture content in GR5 (containing six bodies) remained consistently higher than all the other graves across the study period (June 2016–October 2017) (Figure 9). This is probably associated with the fact that GR5 was deeper than all the other graves. In addition, the comparatively larger number of human cadavers in GR5 inevitably contributed to increased decomposition fluids. It is, however, interesting to note, that despite GR1 and GR2 being the same depth, GR2 (the empty control) was moister than GR1 (with 1 body). The extent to which this increased rainfall influenced decomposition is impossible to define at this date.

**Figure 10.**
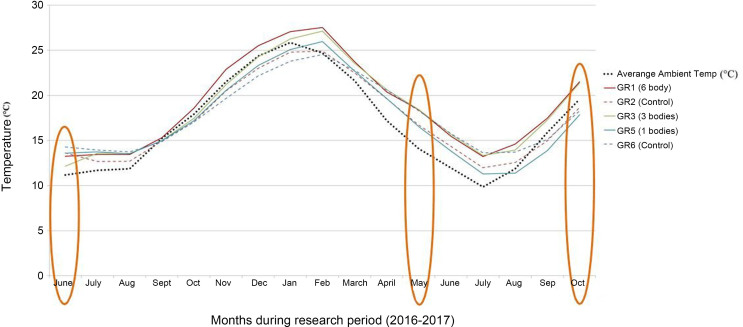
Average monthly ambient temperature compared to average monthly grave temperatures for GR1, GR2, GR3, GR5 and GR6. NB: Orange ovals indicate the months when the LiDAR surveys were completed.

The mean temperatures for all the graves were warmer than the average ambient temperature for the first 3 months (June–September 2016) (Figure 10). For the next approximately 5 months, however, the average temperatures in GR5 and GR6 were lower than the average ambient temperature, after which they become higher again. It is also interesting to note that after about 9 months following the creation of the graves the empty control grave (GR6) was consistently warmer than both the ambient temperature and GR5. At the times of the LiDAR surveys (see Figure 10 highlighted in orange), GR5 (with bodies) was always cooler than GR6 (control) with the ambient temperature only becoming warmer during the third LiDAR survey. Experience has shown that decomposing bodies are warm until the available oxygen in the graves is depleted and the process of putrefaction stagnates (Wright R., Personal communication, 2018) [115]. Thus, in the case of GR5, it appears that the bodies used up all the atmospheric and iron bound oxygen by the about the ninth month post-burial, and consequently, were no longer warmer than the control grave. However, the reason why the control grave (GR6) was consistently warmer than the grave containing bodies (GR5) is unclear.

The results for GR5 and GR6 outlined above are in contrast to those seen in GR1 (one body) and GR2 (control). With the exception of the initial time of burial, the average monthly temperature in GR1 is consistently warmer than GR2 (the control) (Figure 10).

These results suggest that while decomposition in GR1 was occurring, because there was only one body in a relatively shallow grave, the depletion of oxygen as part of the decomposition process was more than likely slower than the depletion in GR5 (with six bodies). Consequently, putrefaction generating heat continued beyond 9 months in GR1. This process has previously been documented in the literature detailing preservation in mass graves: individuals in the middle of the body mass show extremely good preservation (e.g. eyelids) in comparison to those positioned on the edge of the body mass which are skeletonized [19,103].

### Landscape changes

**Figure 11.**
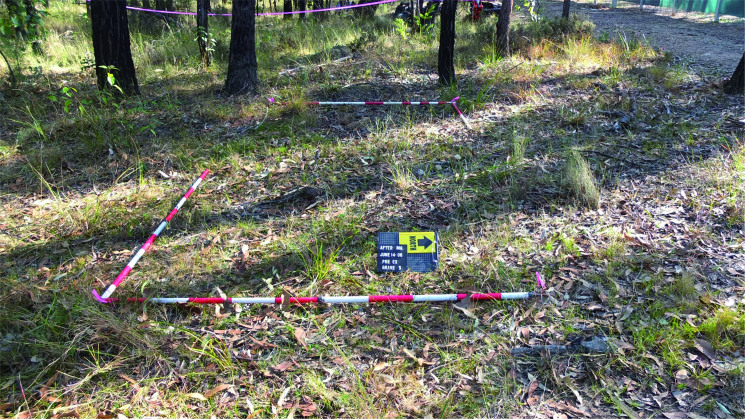
The area designated for the creation of GR5 showing the flat terrain and associated native vegetation (predominantly grasses) and shrubs.

**Figure 12.**
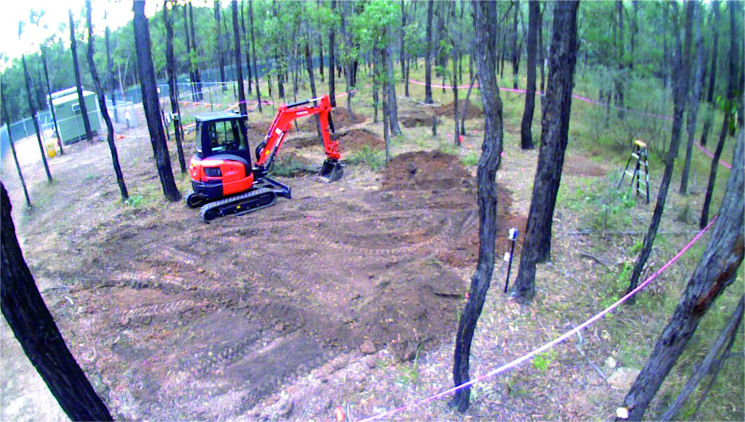
View showing the general area of the experimental graves following backfilling of GR5 (June 2016).

**Figure 13.**
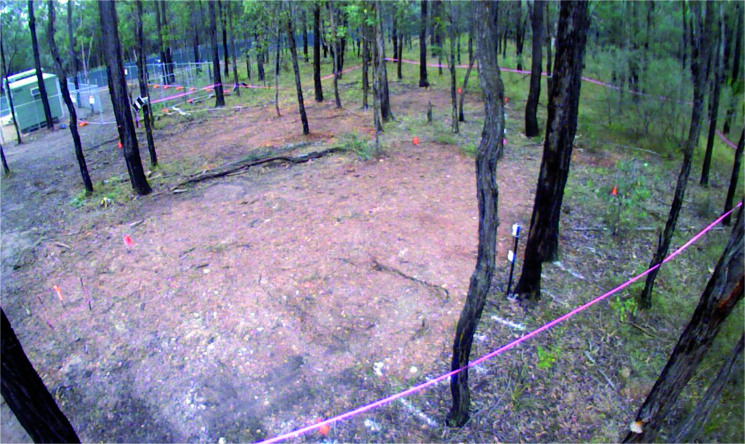
View of GR5 4 days after interment and showing cracking at the edges and slight depression within the deeper area of the grave (June 2016).

**Figure 14.**
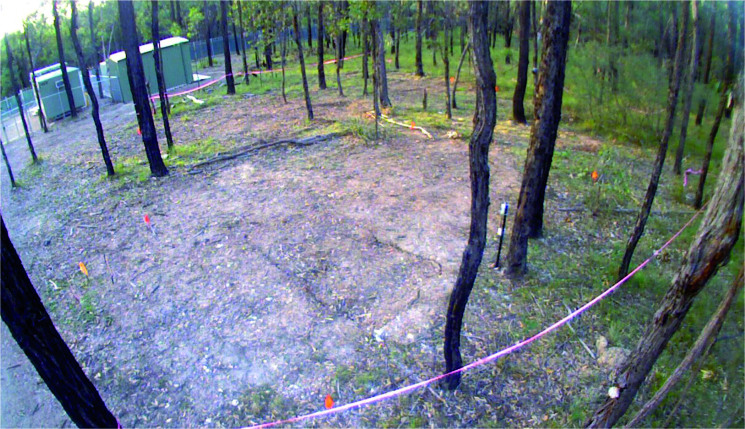
View of GR5 3 months after interment indicating increased cracking to the edges of the grave outline (September 2016).

Prior to the creation of the experimental graves and the controls, the area was relatively flat and covered with native vegetation (predominantly grasses) and shrubs (Figure 11). There was significant overhead coverage provided by the eucalypt woodland. At the completion of interment of the individuals and backfilling the graves (June 2016), time-lapse imagery looking southwest across the site area showed the obvious areas of disturbance (Figure 12). Four days following the interment, GR5 showed cracking around the edges and slight sinkage in the middle of the grave (Figure 13). Three months following internment (September 2016) the grave remained exposed with very minor vegetation growth. Vegetation around the area was relatively lush due to a wet spring. Cracking around the grave cut became more evident (Figure 14).

**Figure 15.**
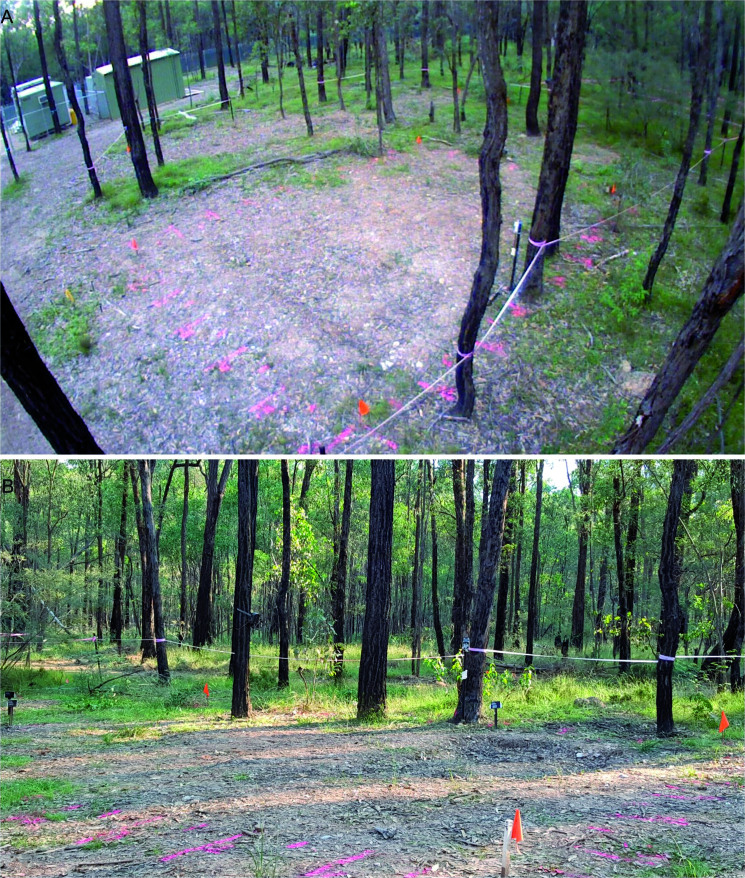
Views of GR5 from the time-lapse camera (A) and looking southwest (B), 11 months after interment indicating an obvious depression and slumping of the backfill (May 2017).

By May 2017 (11 months after internment), the depression within GR5 (presumably associated with decomposition and subsidence/compaction of the backfill) was still evident. However, cracking to the edge of the grave was less obvious (Figure 15).

**Figure 16.**
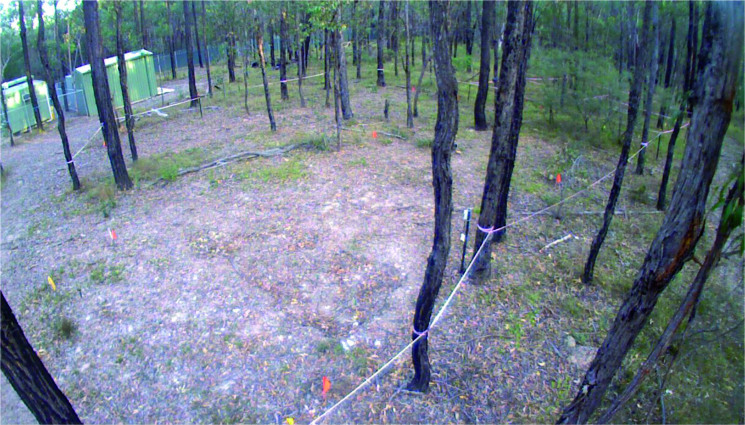
View of GR5 16 months after interment showing increased leaf litter within the obvious grave outline (October 2017).

By October 2017 (16 months following interment), there was increased leaf litter on GR5 but the outline was still relatively visible (Figure 16). While the areas where the graves were present remained sparse of vegetation, the surrounding areas were vegetated.

### Light detection and ranging (LiDAR)

**Figure 17.**
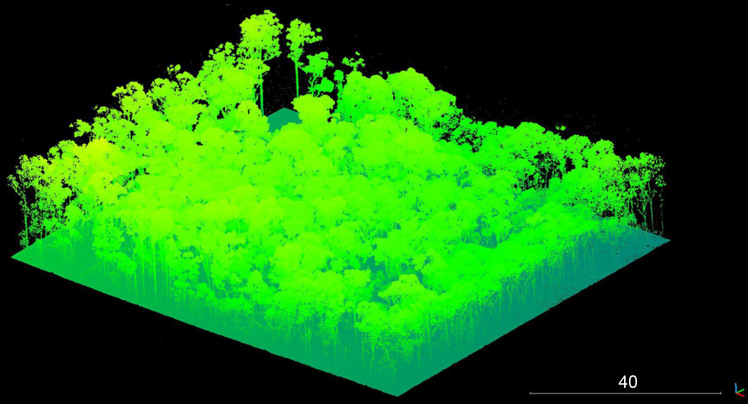
Raw point cloud representing the surveyed site. NB: thick tree canopy.

**Figure 18.**
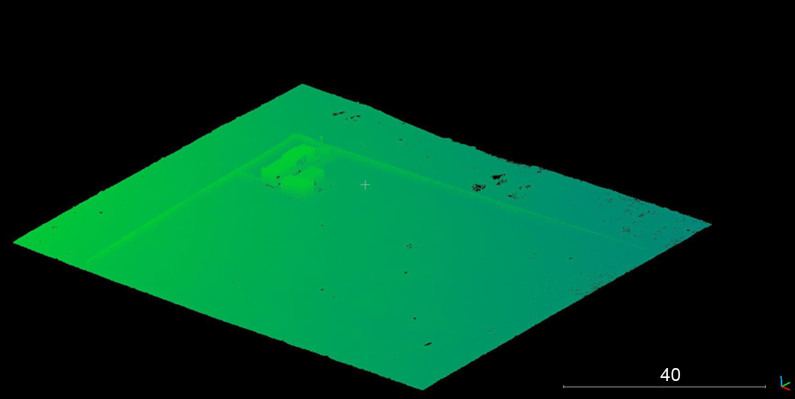
Example of ground model as a result of removal of all vegetation during post-processing.

The initial aerial survey was undertaken to produce a DEM of the area of the site (Figure 17). This formed the basis for all subsequent surveys. While tree canopy was an issue for regular aerial imaging of the site (Figures 1 and 17), the use of airborne LiDAR enabled a digital removal of the tree canopy to obtain a clear view of the underlying terrain (Figure 18). Post-processing of data collected from the two LiDAR surveys facilitated the generation of extremely accurate 3D models of the area.

**Figure 19.**
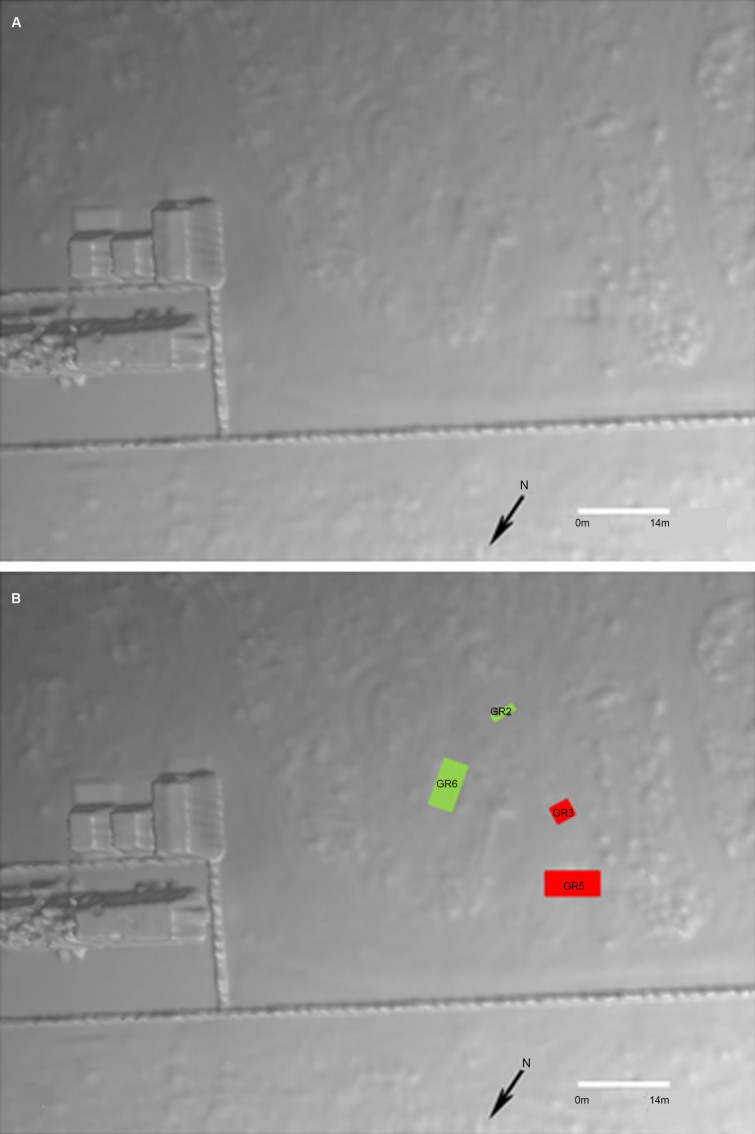
The 3D model following the LiDAR survey in May 2017 (11 months following interment) from which four graves could be identified (A); annotated (B).

From the 3D model developed following the LiDAR survey in May 2017, it was possible to discern anomalies that related to four of the six graves (Figure 19). When compared to the locations of the actual graves it is clear that LiDAR has the accuracy to identify the outline of the grave and the slumping of backfill within the grave itself against the natural undisturbed geology of the area.

**Figure 20.**
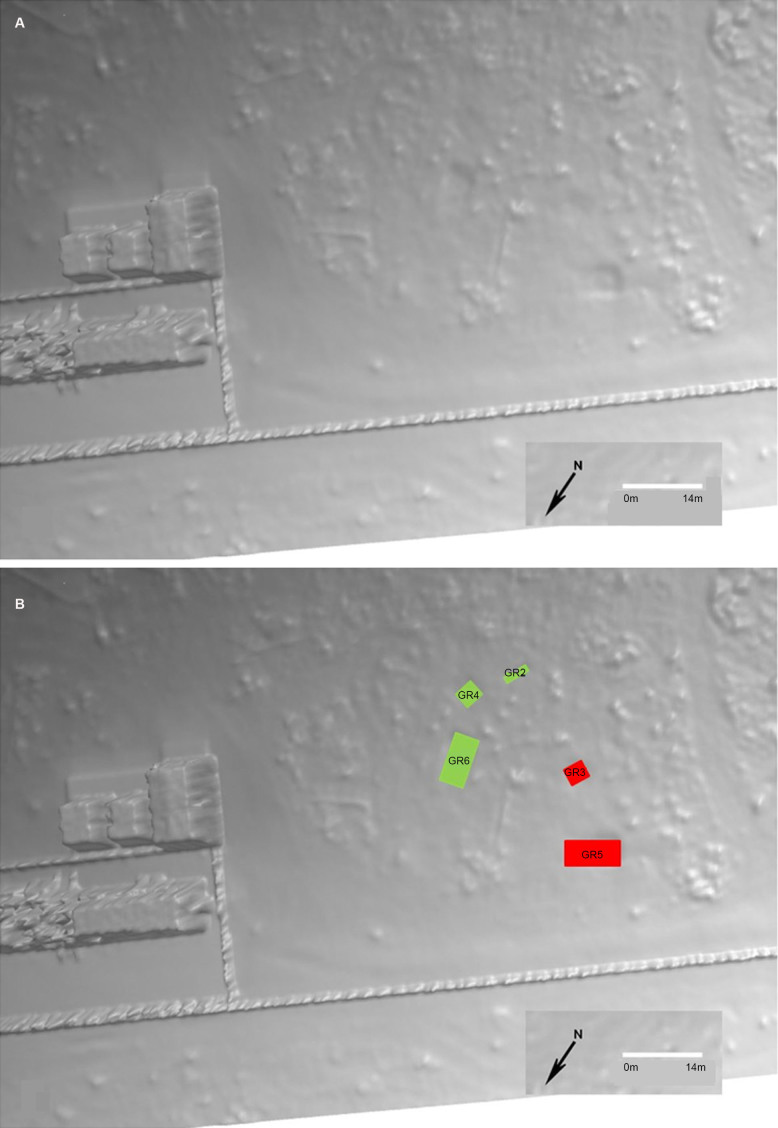
The 3D model following the LiDAR survey in October 2017 (16 months following interment) from which five graves could be identified (A); annotated (B).

The 3D model following the LiDAR survey in October 2017 (16 months following interment) was able to accurately locate an additional grave (GR4 – the control grave) (Figure 20). Interestingly, GR1 (the single human burial) was not identifiable. Further refinement of the LiDAR data may enable this grave to be identified.

## Discussion

While the analysis of all the combined data will be constantly updated until the final exhumation (currently scheduled for 2019), the results of the research to date, a period of 1.5 years from initial burial, are promising. While the experimental and control larger graves and the single control grave remained visible from the air over time, it is interesting to note that the single burial was not detected. While this lack of detection may be related to size (although the control grave was detectable) it may also be related to low topographic contrast with the surrounding ground. While topographic change was not specifically measured in each grave (unlike the study by Corocoran et al. 2018), the differences between the single and larger were visually clear. Once the approximate location of GR1 was revealed to the specialist working with the LiDAR data, an inspection of the point clouds again did not reveal enough evidence that would facilitate detection of the grave. At this stage of the research it is not possible to define whether the grave temperature and moisture data have an influence on the LiDAR analysis.

Once the LiDAR specialist had completed analysis of the data and was subsequently told there were six bodies in one of the graves (GR5), additional GIS analysis was undertaken using a suite of software including Global Mapper, QGIS, Erdas Imagine and Cloud compare. The GIS results indicated that the depression made by GR5 was equivalent to 0.42 m^3^ (at October 2017). Given that the average adult human body measures 0.0711 m^3^ [116] it is interesting to note that the result of dividing 0.42 by 0.0711 estimates the number of interred bodies. At this stage, it is not possible to discern whether the differences in the graves depressions are attributable to differential decomposition and/or subsidence/compaction. However, future work will need to be undertaken to determine if the approach outlined in this article can not only locate the graves, but also estimate the number of buried bodies [117].

Additional methods of detection could include thermal or multispectral and hyperspectral imagery that may also be of use especially where a recent burial is suspected. The temperature data gathered during the research indicate that, as expected, there is a build-up of heat signatures over the graves as a result of decomposition. However, funding to allow infra-red research on the current graves has not been found. Additional LiDAR surveys will be undertaken until mid-2019 (when the three sites containing human cadavers will be excavated and the individuals exhumed^2^) in order to better understand how the different body decomposition stages influence in the grave profile when modelled *via* a point-cloud. Comparing periodic results for the same area of interest will allow differences in topography to be more effectively mapped and for a “grave morphology” database to be developed. It is anticipated that the grave morphology database could serve as a useful tool to train automated classifiers to identify potential graves with no initial human intervention. Finally, the ability to combine LiDAR surveys with simultaneous multispectral analysis of the surrounding vegetation would hopefully determine additional relationships and indexes that can be used together to improve accuracy in mass grave detection.

Currently, the use of chemical signatures of the soil within the graves at AFTER cannot be evaluated. However, emerging technology in the form of hyperspectral sensors which are capable of detecting chemical changes in soils may potentially be able to identify decomposition chemicals on or just below the ground surface as additions to the multi-sensor platform. Finally, although not covered by this research the use of a spectral imaging element within the drone could also be employed. As with the LiDAR surveys currently being undertaken at AFTER there may be unknown or known restrictions to areas of locations where the drone can fly and include areas of military activity or country or state borders.

In conclusion, the final results and analysis of this work will be compared against those already being collected and analysed by colleagues undertaking research in different environments in the northern hemisphere. The research work at AFTER will continue to gather and analyse data for the foreseeable future.

Working towards a multi-sensor platform will need to be undertaken with experts in the relevant technologies. The size of the actual platform if operated from the ground would have to be considered if a drone or similar vehicle is being operated in other than perfect flying and environmental conditions. The drone would also have to be capable of carrying the relevant sensors required for location, at the very least a LiDAR sensor capable of undertaking extreme high-end survey, thermal sensors and multispectral imagery capability. Aerial surveys using an airborne LiDAR sensor have the potential to be employed as a major detector of disturbance and clandestine burial. Post processing to produce detailed 3D models should be seen as one of the major tools available at this time. Careful consideration of landscape changes that take in any footprint of machine activity should be taken.

The preliminary findings of this research are based on the first use of human cadavers in single and mass grave research in a specific environment (sclerophyll forest with sandy silty soil) in the southern hemisphere. While this research represents findings from only one environment, they contribute to augmenting detection techniques. As additional taphonomy research facilities develop, hopefully representative of range of environments, future comparative research based on these findings can be undertaken. Future research should also consider other variables that may influence detection including trauma to individuals and adding different components to backfill, e.g. lime. While the concealment of mass graves is not common in Australia, as a first world country in a region with a wide history of atrocities resulting in the construction and concealment of gravesites, Australia has a responsibility to assist. It is hoped that this article will promote additional discussion on the use of multi-sensor platforms and possibly assist in the development of systems that can be dispatched to any region in the globe where a remote detection of a potential grave can save time, effort, funds and provide a secure method of location.

## Notes

1 It is important to note that there is evidence of bodies being laid out in neat rows in the First World War Holocaust graves. The documented reasons for this relate to the perpetrator’s belief that chaotic burial was an inefficient use of burial space. For example, Schutzstaffel (SS) General Friedrich Jeckeln told a colleague at one of the killing sites in the western Ukraine, “Today we'll stack them like sardines”. This procedure involved victims climbing into the grave and laying down on the bottom. They were then shot. More victims were then made to lie on top of the dead [18].

2 The excavation phase of the project will be used to aid several disciplines that may be applicable for crime scene and humanitarian-based recoveries. These include: forensic archaeology (e.g. mapping and locating gravesites); forensic anthropology (including recovery and analysis of commingled remains), and identification through fingerprints, DNA or clothing where the preservation of skin, DNA, bone, teeth, and finger/toenails, as well as different clothing materials will be assessed. The preservation of other potentially valuable forensic evidence will also be evaluated, including hair, gunshot residue (GSR); and digital information (which will be assessed using specially adapted mobile phones which have been placed in close proximity to the remains). Soil chemistry and its effects on vegetation, stable isotopes and dentition will also be examined.
